# Benzodiazepine partially reverses tonic-clonic seizures induced by thiocolchicoside

**DOI:** 10.1590/1414-431X2021e11771

**Published:** 2022-02-28

**Authors:** D.S. Leitão, A.R. Andrade, N.C.L. Medeiros, M.F.C. Martins, L.O. Ferreira, V.C. Santos, A.O. Hamoy, L.A.L. Barbas, N.A. Muto, V. Jóia de Mello, D.C.F. Lopes, M. Hamoy

**Affiliations:** 1 Laboratório de Farmacologia e Toxicologia de Produtos Naturais, Instituto de Ciências Biológicas, Universidade Federal do Pará, Belém, PA, Brasil; 2 Laboratório de Neuropatologia Experimental, Instituto de Ciências Biológicas, Universidade Federal do Pará, Belém, PA, Brasil; 3 Instituto Federal de Educação, Ciência e Tecnologia do Pará, Castanhal, PA, Brasil; 4 Centro de Valorização de Compostos Bioativos da Amazônia, Universidade Federal do Pará, Belém, PA, Brasil

**Keywords:** Seizure, Thiocolchicoside, Antiepileptic drugs, Electrocorticography, Proconvulsive activity

## Abstract

Seizures are a disorder caused by structural brain lesions, life-threatening metabolic derangements, or drug toxicity. The present study describes the behavior related to proconvulsant activity induced by thiocolchicoside (TCC) in rats and investigates the electrocorticographic patterns of this behavior and the effectiveness of classic antiepileptic drugs used to control these seizures. Forty-nine adult male Wistar rats were used and divided into two phases of our experimental design: 1) evaluation of seizure-related behavior and electrocorticographic patterns induced by TCC and 2) evaluation of the efficacy of classical antiepileptic drugs to control the proconvulsive activity caused by TCC. Our results showed that TCC induced tonic-clonic seizures that caused changes in electrocorticographic readings, characteristic of convulsive activity, with average amplitude greater than that induced by pentylenetetrazole. Treatment with anticonvulsants, especially diazepam, reduced the electrocorticographic outbreaks induced by TCC. The results suggested that TCC caused seizures with increased power in brain oscillations up to 40 Hz and that diazepam may partially reverse the effects.

## Introduction

Seizures are one of the most common neurological emergencies. They usually occur due to an acute injury associated to underlying brain diseases or drug adverse reactions. These acute injuries are often the result of ischemic or hemorrhagic strokes, electrolyte disturbances, and infections. Thus, rapid etiological diagnosis, management, and investigation are essential for a good prognosis and a reduction in brain damage (1,2).

Various medications are known to cause seizures, and extensive research has been conducted to determine the mechanisms that cause these events and to identify individuals that are most susceptible (3). In this context, thiocolchicoside (TCC) is a natural derivative of colchicine and a semisynthetic derivative of colchicoside, a glycoside that occurs naturally in the plant *Gloriosa superba* and is an analog of colchicine (4). This substance has been used for decades, primarily as a non-benzodiazepine muscle relaxant that acts on the central nervous system (5). It also has analgesic and anti-inflammatory properties due to the modulation of chemokine, the production of prostanoids, and the inhibition of the adhesion molecules of the neutrophil and endothelial cells, which may interrupt the inflammation process (4-10).

Although the precise mechanism of TCC is still poorly understood, previous studies have indicated that TCC could act by inhibiting the gamma-aminobutyric acid (GABA) and glycinergic receptors at a central level, inducing muscle relaxation (4,11). Previous studies have suggested a preferential interaction with a cortical subtype of GABA-A receptor that may be related to the convulsing and pro-convulsing actions of this drug (4,11-14).

There are some case reports of TCC-induced seizures in human patients (15-18). Specific experimental or clinical conditions proved that TCC could induce seizures especially in neurological patients with a history of epilepsy (12,15). However, there are few reports of its electrocorticographic (ECoG) activity or possible treatment of TCC-induced seizures with anticonvulsant drugs. It is becoming increasingly necessary to use complementary tools to assist in the clinical and biological assessment of patients with neurological disorders. In this context, electroencephalography is an essential tool for detecting brain activity, especially that related to seizures.

Thus, the present study describes the effects of TCC administration in rats, including seizure-related behavior, compares ECoG parameters with seizure action of pentylenetetrazole (PTZ), and evaluates the efficacy of different anticonvulsant drugs for the management of seizures through ECoG. The main goal of the study was to provide a systematic insight into the development of a therapeutic strategy for the control of TCC-induced seizures.

## Material and Methods

### Animals

The study used 12-14-week-old male Wistar rats (n=56 animals) weighing 300 g (±20 g). Animals were housed in a controlled environment (23±2°C; 12-h light/dark cycle) in standard cages with *ad libitum* access to food and water. All experimental procedures were approved by the proper Brazilian authorities and adhered to the guidelines of the Brazilian National Council for the Control of Animal Experimentation (CONCEA) and the Ethics Committee for the Use of Animals of the Institute of Biological Sciences at the Federal University of Pará (CEPAE-UFPA, Protocol number 101-2015). The experimental data reported comply with the ARRIVE (Animal research: reporting *in vivo* experiments; <https://arriveguidelines.org>) guidelines.

### Drugs

Ketamine hydrochloride was obtained from König Laboratory (Brazil), xylazine hydrochloride was obtained from Vallée laboratory (Brazil), and Coltrax^®^ (thiocolchicoside) and phenobarbital (PBT) were obtained from Aventis Pharma (Brazil). PTZ was purchased from Sigma-Aldrich (USA). All other drugs, such as phenytoin (PHT) and diazepam (DZP), were purchased from União Química (Brazil).

### Experimental design

The study had two phases. In phase 1, after five days of acclimation, the rats were randomly divided into a vehicle-treated (control), PTZ-treated (positive control of induced seizure, 60 mg/kg), or TCC-treated group (n=7 animals per group). This phase was designed to allow for the description of TCC-induced seizure-related behavior (proconvulsant activity) and (ECoG) patterns and their comparison to PTZ-induced responses. In phase 2, after the induction of seizures by TCC, the rats were treated with three anticonvulsant drugs (n=7 animals per group): (i) DZP, 10 mg/kg, (ii) PBT, 10 mg/kg, or (iii) phenytoin (PHT), 10 mg/kg, to evaluate the efficacy of the classical antiepileptic drugs for the control of seizures. All drugs were administered via intraperitoneal (*ip*) injection and solubilized in 0.9% saline. The vehicle group (control) received 0.9% saline at an equivalent volume (mL) to weight (kg) ratio.

### Description of the TCC-induced seizure-related behavior (proconvulsant activity)

The TCC dosage was determined according to a pilot experiment. Doses of 14-, 16-, and 18-mg/kg were tested (12). As the 16-mg/kg dose induced tonic-clonic seizures approximately 15 min after application, this dosage was considered the most suitable for the present study. Following administration of the drug, the latency to changes in behavior was recorded and then the behavioral traits were identified, described based on the existing criteria (19), and classified into four phases: 1) immobility (akinesia), vibrissae lifting, and tail stiffening; 2) head and neck and forelimbs tremors; 3) clonic seizures without loss of the postural reflex; and 4) tonic-clonic seizures with a transient loss of the postural reflex and cyanosis.

### ECoG recordings and data analyses

The ECoG recordings were obtained following the procedure described by Estumano et al. (20). For this, the animals were first anesthetized with ketamine (50 mg/kg, *ip*) and xylazine (10 mg/kg, *ip*). Once the corneal reflex was abolished, the animals were placed in a stereotaxic apparatus. The skull was then exposed and stainless steel electrodes (tip of 1.0 mm in diameter) were placed at the bregma coordinates -0.96±1.0 mm lateral (21). Five days after surgery, the ECoG was recorded using a digital data acquisition system, with offline analysis following the protocol described by Ferreira et al. (22).

Briefly, the animals were kept in individual cages and the electrodes were connected to a digital data acquisition system composed of a high impedance amplifier (Grass Technologies, P511, USA), an oscilloscope (Protek, 6510, USA), and a data acquisition and digitalization board (National Instruments, USA). Data were collected continuously at 1 kHz, at a low pass of 3 kHz and a high pass of 0.3 Hz.

Offline analyses were run using a tool built in the Python programming language (version 2.7, Python Software Foundation, USA), with “Numpy” and “Scipy” libraries used for the mathematical processing and a “matplolib” library used to obtain the graphs and plots. A graphic interface was developed using the PyQt4 library. Spectrograms were calculated using a 256-point Hamming window (256/1,000 s). Each frame of the power spectral density (PSD) was generated with an overlap of 128 points per window. For each frame, the PSD was calculated by the Welch's average periodogram method (22). The frequency histograms were obtained by calculating the PSD of the signal using the 256-point Hamming window without overlap, which yielded a resolution of 1 Hz per bin. Each wave displayed in the PSD is the average from a given set of experiments. The PSD was calculated for each group and the means were shown in individual bins.

Prior to this procedure, the animals were immobilized carefully for 10 min for accommodation to avoid interference. The basal ECoG was then recorded for 15 min, providing the control endpoint for the ECoG analyses. The TCC or PTZ was then administered and ECoG activity was recorded for an additional 15 min. The animals were then euthanized to avoid further distress.

A similar procedure was used to evaluate the TCC-induced seizures and seizure control by the anticonvulsants. In this case, the animals received one of three anticonvulsant agents 5 min after injection of TCC: i) DZP, ii) PBT, or iii) PHT. The ECoG activities were recorded 5 min after the administration of the anticonvulsant drugs and lasted 15 min.

The analyses were performed at a frequency of up to 50 Hz, split into the delta (1-4 Hz), theta (4-8 Hz), alpha (8-12 Hz), beta (12-28 Hz), and gamma (28-40 Hz) bands for the interpretation of the dynamics of seizure development according to Hamoy et al. (19).

### Statistical analysis

The normality and homogeneity of variances were verified using Kolmogorov-Smirnov and Levene's tests, respectively. The results are reported as means±SD, together with F and P values. A P<0.05 level was considered significant for all analyses. Comparisons were made using one-way ANOVA followed by Tukey's test for pairwise comparisons of means. The data were analyzed using GraphPad Prism, version 9 (Graph-Pad Software Inc., USA).

## Results

### Description of TCC-induced seizure-related behavior (epileptogenic activity) and brainwave patterns

**Figure 1:**
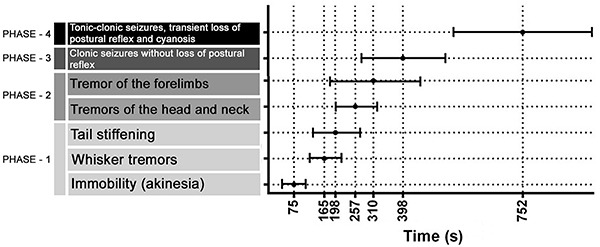
Description of seizure-related behavior induced by thiocolchicoside and latency registration.

After TCC administration, the animals presented the initial symptoms of seizure, such as immobility (akinesia), at 75±32 s, followed by vibrissae lifting (165±45 s), and tail stiffening (198±65 s), which are all components of phase 1 (Figure 1). Phase 2 included head and neck tremors, at 257 s (±63 s), and forelimb tremors, at 310 s (±130 s), which progressed to tonic-clonic seizure without a transient loss of the postural reflex (398±126 s; phase 3), followed by a transient loss of the postural reflex and cyanosis at 752 ± 205 s (phase 4).

**Figure 2:**
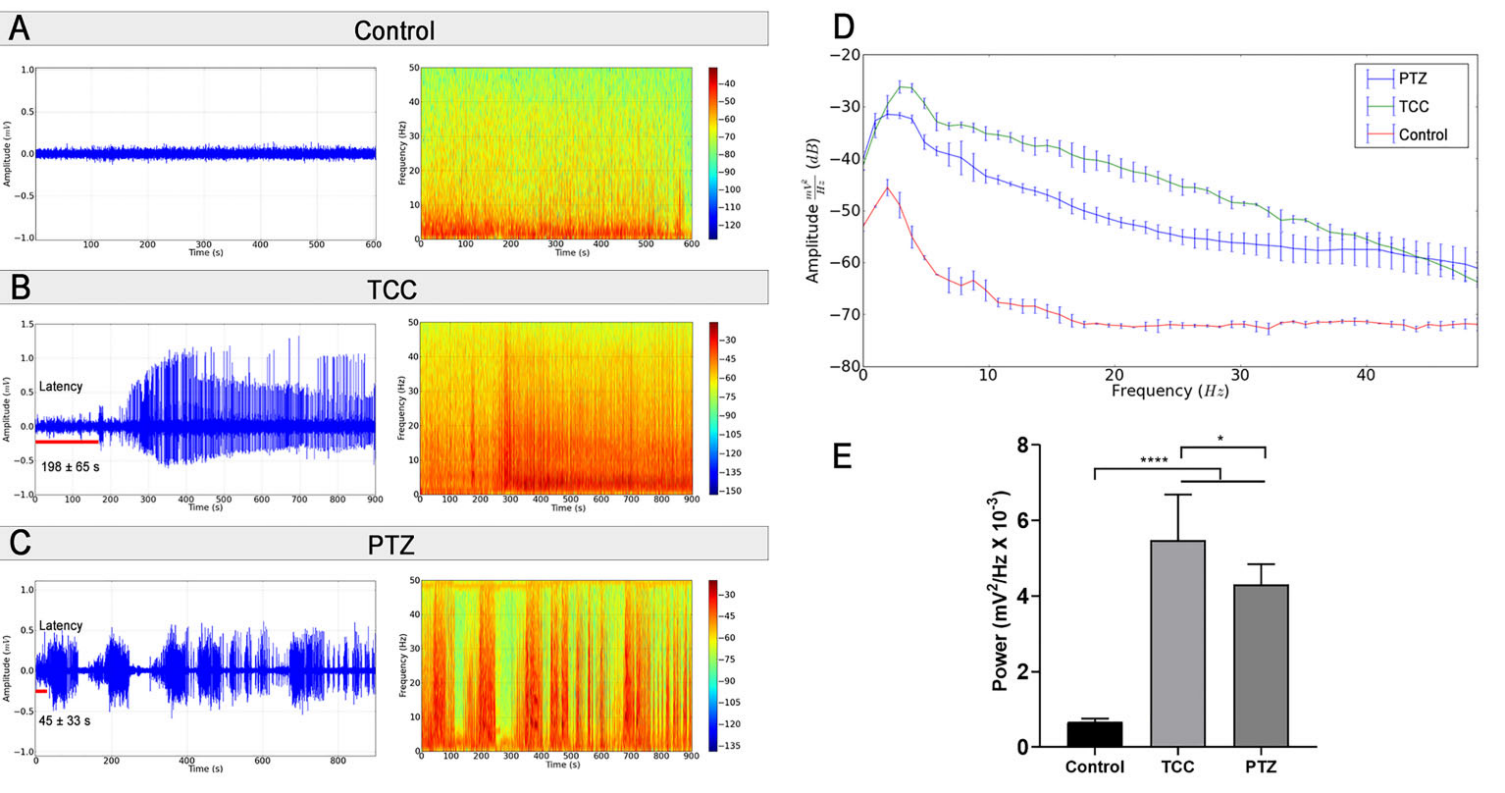
Electrocorticographic recordings of (**A**) control group, (**B**) TCC group, and (**C**) PTZ group. **D**, Representation of the linear frequency distribution between groups with frequency up to 50 Hz. **E**, Representation of the total power linear distribution of cerebral activity. Data are reported as means±SD (n=7 animals/group). *P<0.05 and ****P<0.0001 (one-way ANOVA followed by Tukey's test). TCC: thiocolchicoside; PTZ: pentylenetetrazole.

The ECoG readings of the control group presented low amplitudes with higher levels of energy distributed in frequencies below 10 Hz (Figure 2A). By contrast, the TCC group presented amplitude changes above 1.2 mV (ECoG tracing with cyclic peaks) with an initial latency of 198±65 s and higher energy intensity distributed in up to 50 Hz (Figure 2B). In the PTZ group, changes were observed in the ECoG trace, with cyclic peaks reaching amplitudes of over 0.5 mV, which are characteristic of seizures, with an initial latency of 45±33 s (Figure 2C).

The spectral power distribution revealed greater amplitude in all brainwaves of TCC and PTZ groups compared to the control group (Figure 2D). Significant variation was found among treatments (F_(2,18)_=74.16; P<0.0001) in the distribution of linear frequencies up to 40 Hz (Figure 2E). Both the TCC and PTZ groups presented higher total spectral power relative to the control group (P<0.0001), with a smaller increase observed in the PTZ group, compared to the TCC group (P=0.0284).

The analyses of relative bandpower of brainwaves (delta, theta, alpha, beta, and gamma waves) showed that both TCC and PTZ were able to induce disturbance of all brainwaves, but animals that received TCC presented a greater relative bandpower than animals that received PTZ (Table 1).

**Table 1:** Relative bandpower of brainwaves measured in mV^2^/Hz × 10^-3^.

	Delta	Theta	Alpha	Beta	Gamma
Control	0.09±0.01	0.03±0.002	0.01±0.001	0.01±0.001	0.01±0.002
TCC	1.78±0.16*	1.61±0.15*	0.94±0.08*	1.41±0.06*	0.19±0.03*
PTZ	1.55±0.19*^#^	1.36±0.10*^#^	0.69±0.09*^#^	0.98±0.11*^#^	0.15± 0.03*^#^

Data are reported as means±SD. *P<0.0001 *vs* control group; ^#^P<0.05 *vs* thiocolchicoside (TCC) group (one-way ANOVA). PTZ: pentylenetetrazole.

### TCC-induced seizures altered ECoG patterns and were attenuated by anticonvulsant drugs

**Figure 3:**
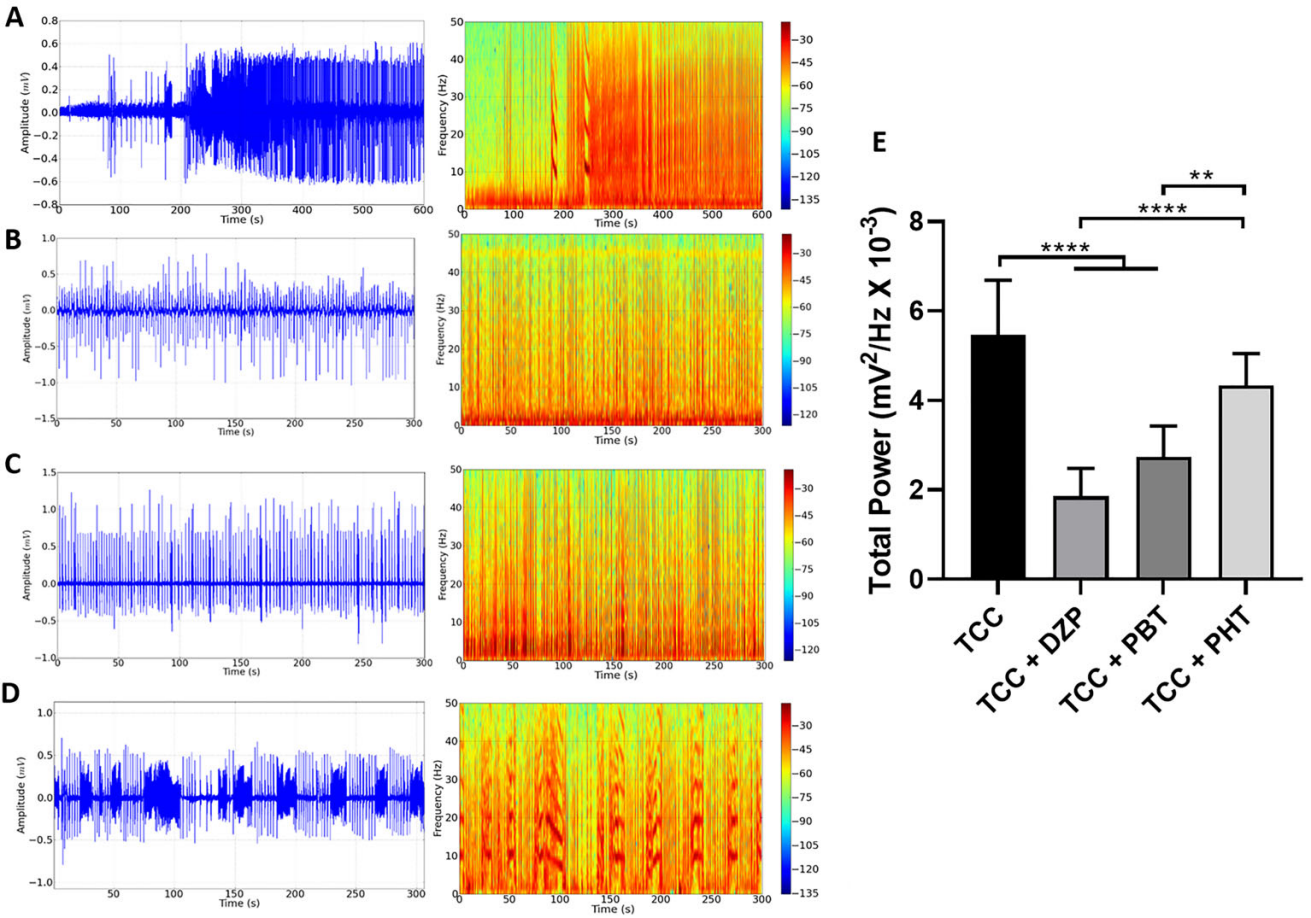
Representation of the total spectral power distribution of (**A**) thiocolchicoside (TCC) group, (**B**) diazepam (DZP) group, (**C**) phenobarbital (PBT) group, (**D**) phenytoin (PHT) group. **E**, Representation of the total power linear distribution of cerebral activity in the groups. Data are reported as means±SD (n=7 animals/group). **P<0.01 and ****P<0.0001, (one-way ANOVA followed by Tukey's test).

After describing the TCC-induced seizures (Figure 3A), we tested whether the anticonvulsant drugs were capable of abolishing or reducing the alterations in the brainwaves provoked by the drug.

DZP decreased seizure activity, with a reduction in the intensity of the cyclic peaks and mean amplitude variation of less than 0.5 mV (Figure 3B). PBT also presented a reduction in the intensity of the cyclic peaks, albeit with amplitude peaking above 0.5 mV (Figure 3C). By contrast, PHT had little effect on the intensity of the cyclic peaks, with both the amplitude and the intensity of the cycles being maintained throughout the whole recording session (Figure 3D).

The total spectral power distribution revealed significant variation among treatments (F_(3, 24)_=25.54; P<0.0001). Thus, only DZP (1.86±0.62 mV^2^/Hz × 10^-3^) and PBT (2.74±0.69 mV^2^/Hz × 10^-3^) were associated with a significant reduction in TCC-induced seizures (5.47±1.22 mV^2^/Hz × 10^-3^; P<0.0001). By contrast, treatment with PHT (4.33±0.71 mV^2^/Hz × 10^-3^) did not have any controlling effect on these seizures (TCC *vs* TCC + PHT, P=0.0825; Figure 3E).

### Relative bandpower profile of brainwaves and treatment of TCC-induced seizures

For a better understanding of anticonvulsant actions, the relative bandpower of oscillations delta, theta, alpha, beta, and gamma were individually analyzed (Table 2).

**Table 2:** Relative bandpower of brainwaves measured in mV^2^/Hz × 10^-3^.

	Delta	Theta	Alpha	Beta	Gamma
	F_(3, 24)_=72.26 P<0.0001	F_(3, 24)_=266.5 P<0.0001	F_(3, 24)_=195.4 P<0.0001	F_(3, 24)_=122.3 P<0.0001	F_(3, 24)_=127.6 P<0.0001
TCC	1.78±0.16	1.61±0.15	0.94±0.08	1.41±0.06	0.19±0.03
TCC+DZP	0.57±0.11*	0.33±0.03*	0.12±0.01*	0.22±0.06*	0.03±0.01*
TCC+PBT	0.96±0.19*^#^	0.51±0.05*^#^	0.24±0.03*^#^	0.67±0.17*^#^	0.07±0.01*^#^
TCC+PHT	1.08±0.16*^#^	0.84±0.09*^#&^	0.48±0.10*^#&^	0.82±0.13*^#^	0.07±0.01*^#^

Data are reported as means±SD. *P<0.0001 *vs* TCC group; ^#^P<0.05 *vs* TCC+DZP group; ^&^P<0.0001 *vs* TCC+PBT group (one-way ANOVA). TCC: thiocolchicoside; DZP: diazepam; PBT: phenobarbital; PHT: phenytoin.

**Figure 4:**
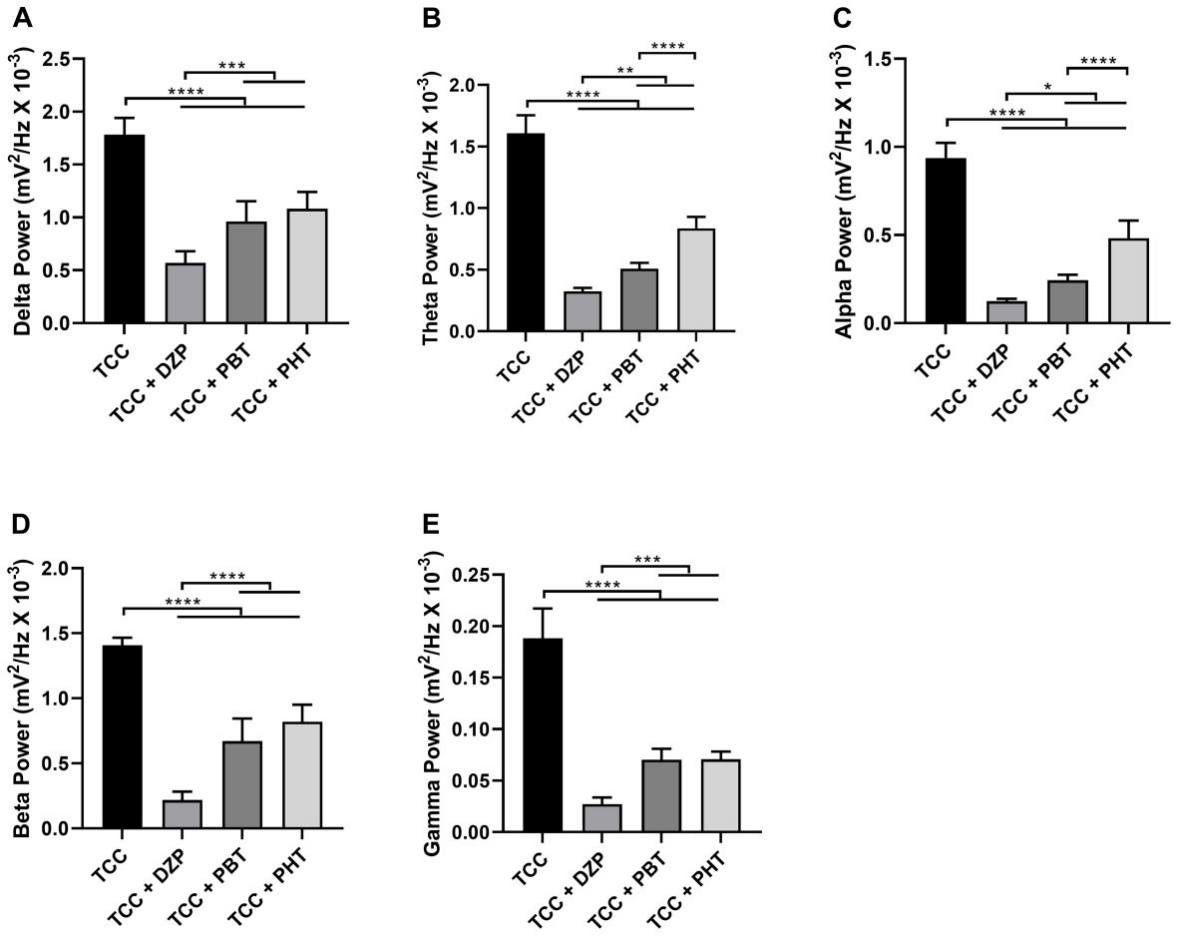
Quantitative linear frequency distribution of the brainwaves in animals submitted to TCC-induced seizures and treated with anticonvulsant drugs. **A**, Delta waves; **B**, theta waves; **C**, alpha waves; **D**, beta waves; and **E**, gamma waves. Data are reported as means±SD (n=7 animals/group). *P<0.05, **P<0.01, ***P<0.001, and ****P<0.0001 (one-way ANOVA followed by Tukey's test). TCC: thiocolchicoside; DZP: diazepam; PBT: phenobarbital; PHT: phenytoin.

For delta, beta, and gamma relative bandpower, all anticonvulsants reduced the oscillations of brainwaves compared with TCC (TCC *vs* TCC + anticonvulsants drugs, P<0.0001 for all comparisons to these brainwaves). DZP was more efficient than PBT and PHT in controlling the seizures induced by TCC (*vs* TCC+PBT and TCC+PHT, P<0.001 for all comparisons to these brainwaves) and no statistical differences were observed between PBT and PHT groups (P>0.05 for all comparisons to these brainwaves; Figure 4A, D, E).

All anticonvulsants reduced the theta and alpha relative bandpower compared with TCC (TCC *vs* TCC + anticonvulsants drugs, P<0.0001 for all comparisons to these brainwaves). DZP was the most effective treatment (*vs* TCC+PBT and TCC+PHT, P<0.05 for comparisons to theta and alpha brainwaves), whereas PHT was the least effective treatment (*vs* TCC+PBT, P<0.0001 for comparisons to theta and alpha brainwaves; Figure 4B, C).

## Discussion

A well-balanced interaction of inhibitory and excitatory neuronal activity is a basic prerequisite for physiological function of the central nervous system. In general, when disordered excitatory triggers predominate, convulsive events may occur. The main mechanisms involved are related to hyperactivity of the excitatory amino acid systems, impaired inhibition mediated by GABA-A receptors, and changes in the intrinsic properties of the neuronal membranes (1).

The main mechanisms involved in decreasing seizure threshold are related to the imbalance between excitatory and inhibitory pathways that are mediated by neurotransmitters. Thus, processes that impair the activity of inhibitory mediators cause excitability that results in seizures (1,19).

Our results showed that TCC affected behavior, producing tonic-clonic activity approximately 12 min after the beginning of the application. Furthermore, the rats treated with TCC had an ECoG tracing and brain wave higher than the positive control for seizures (PTZ), which showed that TCC had a seizure pattern and it could be used for a seizure model.

Previous studies showed that TCC was able to produce seizures in rats that already had some type of minimal cerebral lesions of the dura mater and arachnoid membranes or in the blood-brain barrier, but without development of seizures in animals without previous injuries. Sechi et al. (12) reported that TCC is able to produce seizures only in rats with damaged meningeal membranes, but our results were different, as in this study we showed the development of tonic-clonic seizures in animals without previous brain damage. This corroborated our data that TCC can produce convulsive effects in healthy individuals.

There are some published case reports of TCC-induced seizures in human patients. One of these studies showed TCC-induced seizure following disturbances of the blood-brain barrier (BBB), which indicates that TCC should be avoided in patients with BBB disruption (15). A second study reports a patient with a diagnosis of cerebral amyloid angiopathy, which disrupts the BBB, and the association with TCC treatment for muscle contracture and pain resulted in seizures (17). A third study involved a healthy 3-month-old patient who had a seizure after her mother had taken TCC while breastfeeding (16). Together, these case reports indicate that TCC should be used with caution in the treatment of patients with brain injuries or epilepsy. However, the mechanism of action of TCC is still not completely understood.

Furthermore, there are few reports showing quantitative electrocorticography in seizures caused by TCC. Our results showed an important increase in the total power associated with an increase in all analyzed frequency bandpower. This corroborated the findings of De Riu et al. (15) of a patient with a TCC-induced seizure and an increase in theta wave, accompanied by mild focal background abnormalities, which were more evident in frontotemporal regions.

Although the mechanism of action is not yet fully understood, some studies suggest that TCC may be related to glycine receptors in some brain regions, such as the brainstem and spinal cord (23). These interactions may be responsible for the therapeutic effects of the medication, in this case myorelaxant activity, but how this agonistic interaction may induce seizures is not known.

Pentylenetetrazol is a non-competitive antagonist of the GABA-A receptors (24) and a valuable pharmacological model for the study of seizures because it is inexpensive and easily reproduced (25). Herein, we showed that TCC at 16 mg/kg was more potent for the induction of seizures than PTZ, corroborating the pro-convulsive potential of TCC described in previous studies (4,12). In our study, the animals with seizures caused by TCC had higher powers in the delta, theta, alpha, beta, and gamma frequency bands compared to the group treated with PTZ (Table 1), indicating greater brain activity. Among the bandpowers analyzed in TCC-induced seizures, the delta and theta bandpowers presented greater activity, and the result was consistent with the literature, which reports that low-frequency brain rhythms are often associated with excessive electrical activity and brain damage (26).

Although both DZP and PBT reduced total electrical brain activity (total power, Figure 3E) during the TCC-induced seizure and all anticonvulsant drugs reduced the relative bandpower of brain oscillations (Figure 4), DZP showed the greatest reduction in frequency changes in the brainwaves attributed to seizures caused by TCC. DZP is part of the initial treatment protocol for seizures as a first-line drug and its use has proven to be important in containing seizures in different scenarios, whether induced by trauma or medications (27).

In summary, we have provided a description of the behavior and ECoG characteristics of TCC-induced seizures in the rat cortex. TCC-induced seizures evolve to tonic-clonic seizures showing greater latency and brain oscillations compared to the PTZ model. Furthermore, we suggest that TCC can be used as an acute seizure model for experimental studies. Nonetheless, further studies are needed to define the mechanism of action underlying the seizure effect of this drug.
